# Thermionic injection analysis in germanium nanowire Schottky junction FETs by means of 1D and 3D extraction methods

**DOI:** 10.1039/d4na00957f

**Published:** 2025-02-18

**Authors:** Raphael Behrle, Aníbal Pacheco-Sanchez, Sven Barth, Walter M. Weber, Masiar Sistani

**Affiliations:** a Institute of Solid State Electronics, TU Wien Vienna 1040 Austria masiar.sistani@tuwien.ac.at; b Departament d'Enginyeria Electrònica, Universitat Autònoma de Barcelona Bellaterra 08193 Spain; c Departamento de Electrónica y Tecnología de Computadores, Universidad de Granada Granada 18071 Spain; d Physics Institute, Goethe Universität Frankfurt Frankfurt am Main 60438 Germany

## Abstract

Schottky barrier field-effect transistors (SBFETs) are a promising family of devices suitable for realizing “Beyond CMOS” paradigms. As the SBFET device operation is strongly dependent on the metal–semiconductor junction properties, it is important to extract and understand the activation energy to inject charge carriers into the semiconductor channel. In this regard, the three-dimensional (3D) thermionic emission (TE) and the one-dimensional (1D) Landauer–Büttiker (LB) theory are among the most sophisticated methods. Here, both methods are used to analyze the charge carrier injection capabilities of Al–Ge–Al nanowire (NW) heterostructure SBFETs. While the 3D TE model underestimates the activation energy *E*_a_ in strong accumulation, at the intrinsic off-point, where merely TE contributes to charge carrier transport, both models provide reasonable values close to the theoretically expected Schottky barrier height. Analyzing the underlying mathematical models of 3D TE and 1D LB reveals a quadratic and linear increase in TE depending on temperature, respectively. Moreover, until now effects on the *E*_a_ originating from the 1D nature of the proposed device were rarely investigated in NW transistors. This comparison contributes to a better understanding and the advancement of SBFET devices and circuit technologies.

## Abstract 2

The carrier transport in Ge-based nanowire transistors is analyzed by temperature-dependent electrical measurements and discussed in terms of three-dimensional thermionic emission and one-dimensional Landauer–Büttiker theory.

## Graphical abstract

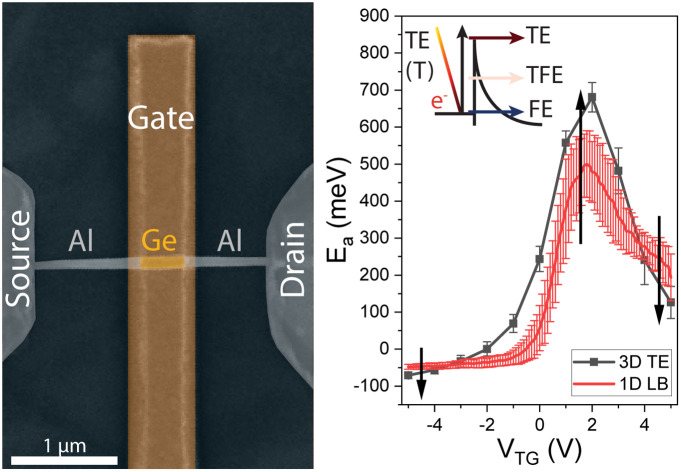

## 1 Introduction

Recently, numerous emerging nanoelectronic and quantum applications have been proposed based on Schottky barrier (SB) field-effect transistors (SBFETs) comprising axial metal–semiconductor–metal heterostructures.^1,2^ Such devices rely on the potential modulation across the active region and in particular at the metal/semiconductor junctions. In this respect, bottom-up grown semiconductor nanowires (NWs) are a remarkable prototyping platform inherently providing high surface smoothness, an ultrathin-body as well as quasi-one-dimensional (1D) nature.^3,4^ Aligning a gate stack atop a metal–semiconductor junction allows the filtering of the charge carrier type and modulating the charge carrier concentration in the semiconductor by tuning the energy bands, *i.e.*, bending them down- or upwards.^5^ This enables promising applications from reconfigurable FETs (RFETs),^6^ and source-gated transistors^7^ to surface plasmon detectors^8,9^ and Josephson junction FETs.^10,11^ In this regard, the properties of the metal–semiconductor junctions play a major role in the overall charge transport mechanism. To characterize the underlying mechanism and efficiency of charge carrier injection, the extraction of the activation energy *E*_a_ has proven to be a useful tool. In contrast to the SB height, which merely considers thermionic emission (TE), *E*_a_ also takes field emission (FE) into account and considers the entire device transport path. Notably, SB extraction generally requires ohmic access to the semiconductor region not available in the device types listed above. Recently, all-optical and non-invasive techniques were introduced to investigate thermionic electron injection^12^ and to analyze the transient absorption spectra.^13^ Nevertheless, in the scope of device or circuit realization, *E*_a_ plays a crucial role, as it physically describes the required minimum energy to inject a considerable amount of charge carriers into the semiconductor. In this respect, metal–Ge junctions have shown different Fermi level pinning, depending strongly on the Ge surface states as reported by Nishimura *et al.*^14^ In the latter study, a dedicated SB (*via* capacitance–voltage and three dimensional (3D) TE models) for electrons was obtained, whereas the barrier for holes is minimal due to the Fermi level pinning being close to the valence band in all investigated metal–Ge junctions.^14^ As a consequence, holes typically experience a more efficient injection in comparison to electrons, resulting in some cases in quasi-ohmic junctions.^15,16^ An important application of such transparent junctions in Al–Ge heterojunction devices are superconductor-semiconductor devices,^17^ as, for example, those used in Al–Ge–Al heterojunction based Josephson junction FETs.^11,18^ The semiconductor dimensionality has been rarely considered in the characterization of the above-mentioned junctions since most of them deal with bulky materials. However, when dealing with 1D semiconductors, such as Ge NWs, where quasi-ballistic transport is expected, the conventional parameter extraction methods developed for 3D materials need to be revisited in order to adapt them to or substitute them for novel methodologies considering more appropriate device physics.^19^ In this work, the 3D TE and 1D Landauer–Büttiker (LB) theory are analyzed, compared, and used to extract the activation energy *E*_a_ of the charge transport in NW-based Al–Ge–Al SBFETs.

## 2 Results and discussion

**Fig. 1:**
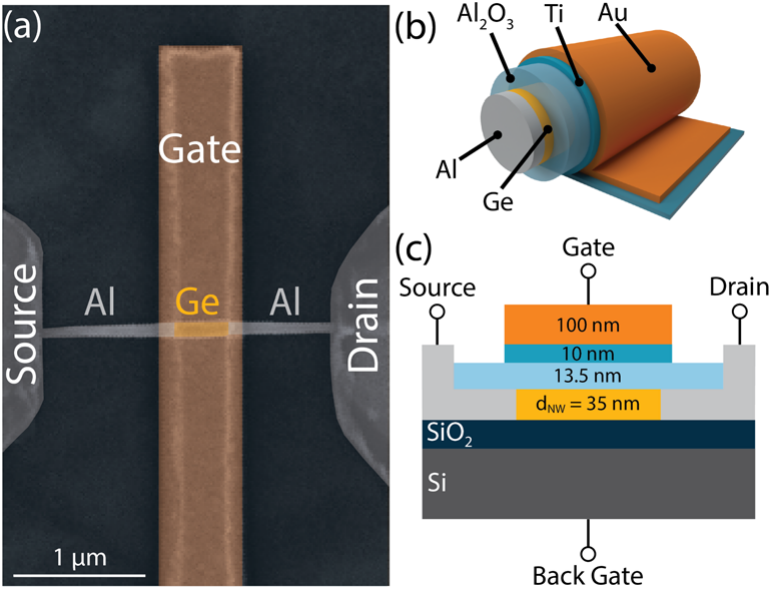
(a) Colored SEM image of the proposed Al–Ge–Al NW-based SBFET. (b) 3D schematic of the NW heterostructure and the device's Ω-shaped top-gate architecture. (c) Axial cross-section with respect to the NWs of the material stack. Note that dimensions are not in scale.

The investigated SBFET devices are based on nominally undoped Ge NWs grown on a Ge (111) single crystal substrate by the vapor–liquid–solid method using Au catalyst particles and a diphenylgermane precursor for Ge.^20^ The Ge NWs exhibit a nominal diameter of *d*_NW_ = 35 nm, which is close to the Ge exciton Bohr radius 
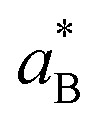
 (Ge: 24.3 nm).^21^ Thus, the 1D density of state confinement needs to be considered. The used precursor enables a NW surface termination with phenyl ligands, which leads to a reduction of native Ge-oxide formation and associated, but undesired, influences of interface states.^15,20,22^ After their growth, the NWs were passivated by a 13.5 nm thick Al_2_O_3_ shell by atomic layer deposition, which later also acts as the SBFET's gate oxide. In the next step, the passivated NWs were transferred onto a p-Si substrate with a 100 nm thick thermally grown SiO_2_ layer on top. Afterwards, Al contacts were fabricated to the Ge NWs by using e-beam lithography (EBL), HF etching, Al sputter deposition and lift-off techniques. To obtain the desired metal–semiconductor–metal heterostructure, a rapid thermal annealing process at 673 K was applied to initiate the Al–Ge solid-state atomic exchange mechanism, which typically results in abrupt Al–Ge junctions with nominal Ge segment lengths of ≈500 nm.^23,24^ In the last step, Ω-shaped Ti top-gates, with Au bond pads covering the Ge channel and metal–semiconductor junctions were fabricated by EBL, evaporation, and lift-off techniques, finally resulting in the SBFET illustrated in the scanning electron microscopy (SEM) image in Fig. 1a. Additionally, Fig. 1b and c show a 3D model and an illustration of the obtained material stack, respectively.

**Fig. 2:**
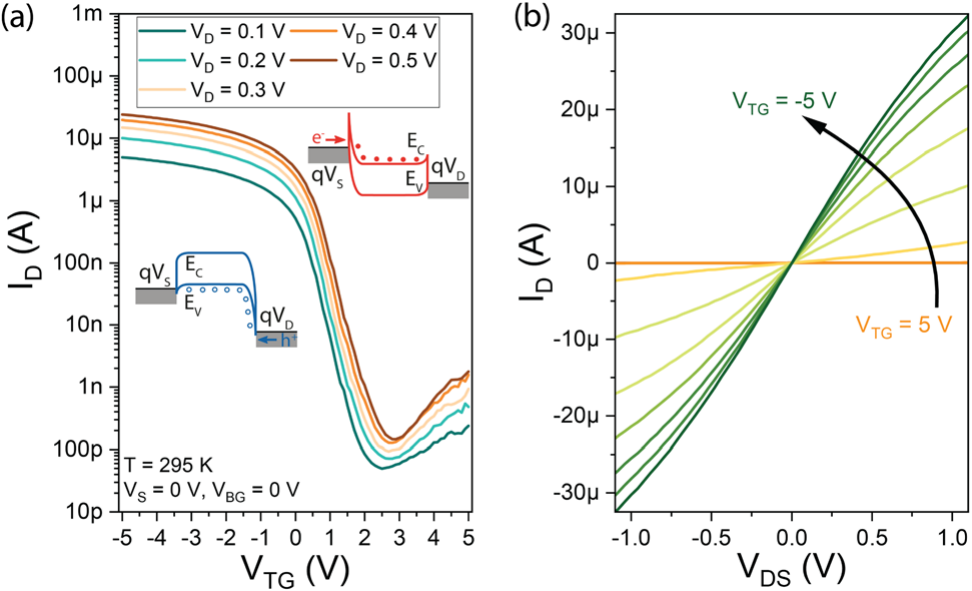
(a) Ambipolar transfer characteristics of a representative SBFET at different bias voltages *V*_D_ ranging from 0.1 V to 0.5 V. The insets show simplified band diagrams for electron injection (red; positive *V*_TG_) and hole injection (blue; negative *V*_TG_). (b) Linear visualization of the output *I*/*V* characteristics at different top-gate voltages *V*_TG_ ranging from 5 V to −5 V. At low |*V*_DS_| a linear trend for *V*_TG_ < 0 V is evident, indicating quasi-ohmic junctions for hole injection.

Fig. 2a shows transfer characteristics of the considered Al–Ge–Al NW-based SBFET at different *V*_D_ biases ranging from 0.1 V to 0.5 V. Note that, throughout this study the back gate voltage *V*_BG_ is set to 0 V. Analyzing the transfer characteristics reveals the dominant p-type characteristics of Al–Ge–Al SBFETs, which are caused by Fermi level pinning close to the valence band edge of Ge.^14^ As a consequence, the injection of holes is more efficient due to a much lower SB in comparison to that of electron injection.^15^ Therefore, an electron on-current *I*^n^_on_ of 1.8 nA (0.05 μA μm^−1^) is provided at *V*_TG_ = 5 V and a hole on-current *I*^p^_on_ = 23.7 μA (677 μA μm^−1^) at *V*_TG_ = −5 V, both reported at *V*_D_ = 0.5 V. Note that values in brackets are normalized on-state currents to the nominal *d*_NW_. This strong gating tunability of 5 orders of magnitude in the hole dominant regime, considering an off-current *I*_off_ = 152 pA, is achieved by efficient electrostatic tuning of the energy bands as illustrated in the insets of Fig. 2a. Consequently, applying *V*_TG_ ≳ 3 V (at *V*_D_ = 0.5 V) leads to predominant thermionic emission of electrons over the barrier until the applied *V*_TG_ bends the potential bands downwards, so that the SB is thin enough to allow tunneling-dominated electron injection into the conduction band *E*_C_. In contrast, a *V*_TG_ ≲3 V (at *V*_D_ = 0.5 V) leads to an upwards bending of the bands. As the SB for holes is already small, *i.e.*, TE dominates for holes, further band bending induces SB thinning, and an enhanced injection of holes is achieved. This behavior is also evidently observable in the linear representation of the output characteristics illustrated in Fig. 2b, which confirms a linear *I*/*V* at low |*V*_DS_|, demonstrating quasi-ohmic contacts.

In terms of charge carrier injection thermal effects need to be considered, which will also build the base for the investigations of the activation energy extraction below. Increasing the temperature leads to a significant increase in charge carriers overcoming the SB due to their thermally increased energy. A simplified schematic of the charge carrier injection mechanisms is provided later in Fig. 4a, where the different injection mechanisms are further elaborated on.

**Fig. 3:**
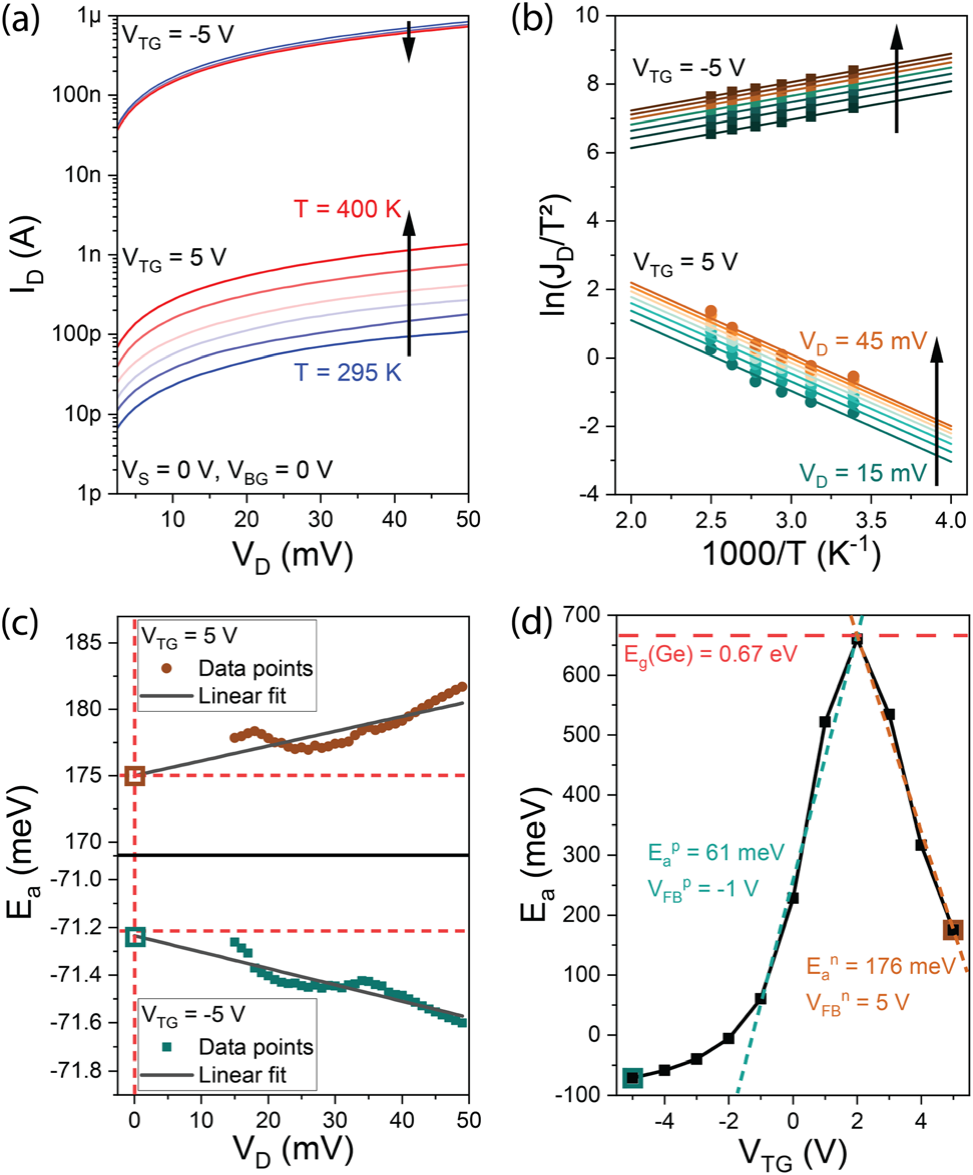
(a) Temperature-dependent output characteristics in strong electron and hole accumulation at *V*_TG_ = 5 V and −5 V, respectively. (b) Arrhenius plots of the two corresponding points for selected bias voltages *V*_D_. The *x*-axis has been multiplied by a factor of 1000 for plotting purposes. (c) Linear fitting of the *V*_D_-dependent *E*_a_ values to *V*_D_ = 0 V at the two selected *V*_TG_ biases. (d) *V*_TG_-Dependent *E*_a_ values with indicated *E*^n/p^_a_ and *V*^n/p^_FB_ values for electrons and holes.

In the first step the activation energy *E*_a_ is investigated by (3D) TE theory, which in general is defined by the model shown in eqn (1).^25^1
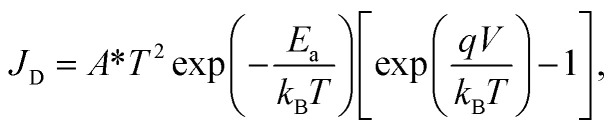
where *J*_D_ is the total current density in A m^−2^ with the NW cross-section area being 1.45 × 10^−11^ cm^2^, A* is the effective 3D Richardson constant, *T* is the corresponding absolute temperature, *q* is the elementary charge, *E*_a_ is the activation energy, *k*_B_ is the Boltzmann constant and *V* is the drain/source potential (here: *V*_D_). Importantly, in SBFETs, *V*_D_ and *V*_TG_ directly influence the activation energy necessary to inject charge carriers.^5^ When using eqn (1) particular boundary conditions with respect to *V*_D_ need to be considered. First, *V*_D_ can merely be investigated in the linear regime of the underlying output *I*/*V* characteristics as well as considering *V*_D_ > *k*_B_*T*/*q* (>25 mV at *T* = 295 K), *i.e.*, ensuring charge carrier injection by TE. Moreover, in an experimental setup, it cannot be differentiated if the injected charge carriers originate from TE and/or FE. Consequently, in strong charge carrier accumulation – induced by strong band bending (|*V*_TG_| ≫) and therefore thinned SBs – FE is the dominant injection mechanism, where TE models fail. With respect to the experimental extraction of *E*_a_, it needs to be considered that the 3D Richardson constant A* is unknown. Therefore, *E*_a_ is obtained by measuring the output *I*/*V* characteristics over a given temperature range (here: *T* = 295 K to 400 K) (*cf.*Fig. 3a) to extract *V*_TG_-dependent *E*_a_ values. Next, an Arrhenius plot (*cf.*Fig. 3b) is generated, which gives a linear trend with slope *m* = −*E*_a_/*k*_B_ + *qV*_D_/*k*_B_. Moreover, considering the term exp[*qV*_D_/(*k*_B_*T*)] ≫ 1 [*cf.*eqn (1)], eqn (2) is derived and shows the mathematical relation to obtain Fig. 3b.2
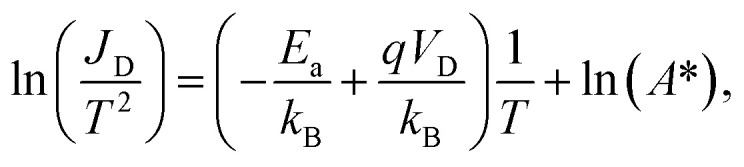
which follows a linear 1/*T* dependence of the left-side term. Finally, eqn (3) shows the final expression to obtain *E*_a_ from the slope *m* extracted from Fig. 3b.

3 *E*_a_ = *qV*_D_ − *mk*_B_

Considering Fig. 3c, for each *V*_D_ data point, a dedicated *E*_a_ can be deduced, which allows a linear extrapolation to *V*_D_ = 0 V to extract *E*_a_ depending on *V*_TG_. Therefore, *E*_a_ = −*mk*_B_ holds. Performing the same methodology for *V*_TG_ = 5 V to −5 V in 1 V steps finally results in Fig. 3d, which shows the *V*_TG_-dependent *E*_a_ of the proposed SBFET.

Remarkably, on analyzing Fig. 3d, negative *E*_a_ values are obtained in strong hole accumulation, which further impact the output and Arrhenius plots (*cf.*Fig. 3a and b). Increasing the temperature results in a decrease in the hole current (*V*_TG_ = −5 V), whereas the electron current (*V*_TG_ = 5 V) increases, due to thermally excited charge carriers. This mechanism is also visible in the positive slope *m* of the Arrhenius plot (*cf.*Fig. 3b). Consequently, due to the low value obtained for *E*^p^_a_, we assume that the channel resistance is the main contributor in limiting the current and that the Al–Ge junctions are transparent for holes.^15^ However, at the intrinsic off-point (*V*_TG_ ≈ 2 V), a value close to the band gap of bulk Ge is reached, where merely TE contributes, demonstrating the suitability of the 3D TE model in this regime. Finally, activation energies *E*_a_ and flat band voltages *V*_FB_ for n- and p-type conduction are approximated by a linear fit in the linear regime of the *V*_TG_-dependent *E*_a_ (*cf.*Fig. 3d). Remarkably, this extrapolation corresponds to the subthreshold operation regime, where TE dominates. It can be assumed that a higher resolution of the *V*_TG_ step-width would be required for a more accurate estimation. Remarkably, 1D LB also tackles this issue as demonstrated next.

1D transport at the subthreshold regime in a NW-based SBFET can be described in the context of the LB model^26^ by considering quasi-ballistic conduction restricted to the first sub-band. Hence, after some approximations involving Boltzmann statistics, the drain current *I*_D_ in quasi-ballistic SBFETs due to TE is obtained according to^27,28^4
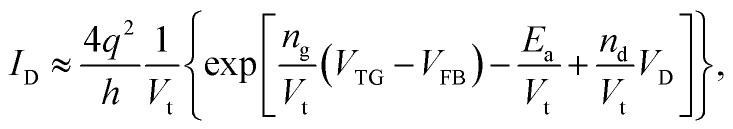
where *V*_t_ = *k*_B_*T*/*q* is the thermal voltage, *h* is Planck's constant, *n*_g_ and *n*_d_ are gate and drain coupling coefficients, respectively, and *E*_a_ is an effective energy potential barrier over which pure TE is expected corresponding to the activation energy described above. Note that both *V*_TG_ and *V*_D_ dependencies are considered explicitly in *I*_D_ by the LB model in contrast to TE theory.

**Fig. 4:**
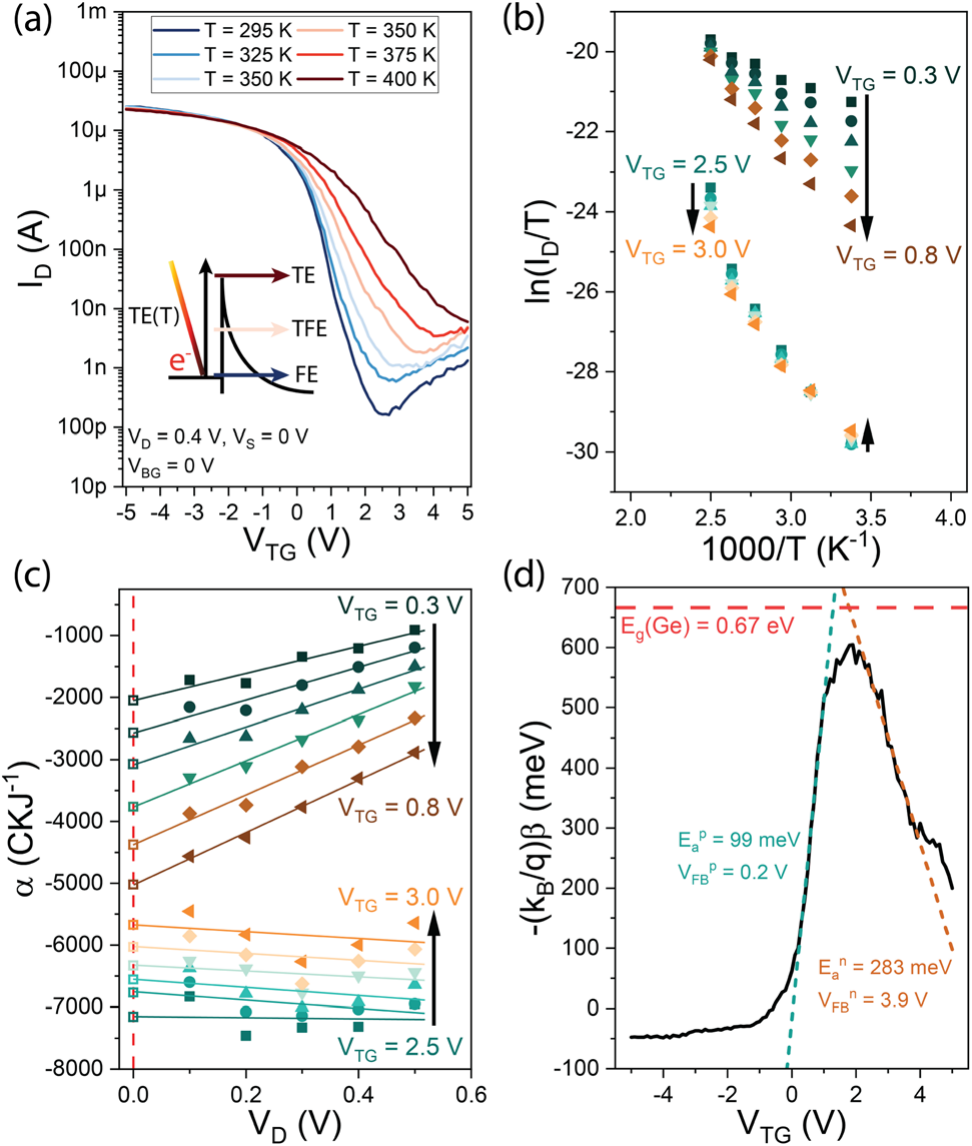
(a) Temperature dependent transfer characteristics at *V*_D_ = 0.1 V. (b) Arrhenius plot required for 1D LBM extraction at *V*_D_ = 0.1 V. The *x*-axis has been multiplied by a factor of 1000 for clarity. (c) The slope of Arrhenius plots at different *V*_D_. (d) Activation energy plot from where the effective SB heights are obtained at *V*_FB_ with *β* = *n*_g_(*V*_TG_ − *V*_FB_) − *E*_a_. (b and c) *V*_TG_ data corresponding to the n- and p-type subthreshold regimes.

Based on eqn (4), an Arrhenius-like equation can be obtained after some rearrangements such as5
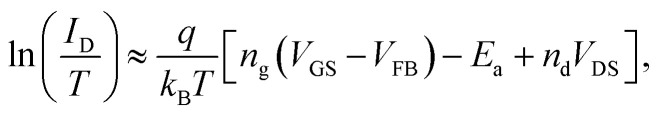
and *E*_a_ can be extracted by applying the following methodology: (i) obtain the experimental transfer device characteristics at different *V*_D_ and different *T*, *cf.*, Fig. 4a; (ii) map an Arrhenius plot, *i.e.*, ln(*I*_D_/*T*) *vs.* 1/*T* (*cf.*Fig. 4b); (iii) extract the slope from it, *i.e.*, *α* = *∂*[ln(*I*_D_/*T*)]/*∂*(1/*T*) for the different *V*_TG_ and at each *V*_DS_ (*cf.*Fig. 4); (iv) by obtaining the value of *α*|_*V*_DS_→0_ for each *V*_TG_ an energy function related to the bias and *E*_a_ is obtained, *i.e.*, *E*_a_ = −(*k*_B_*β*)/*q* (*cf.*Fig. 4d); (v) *E*_a_ corresponding to the energy potential barrier for a specific carrier is obtained at *V*_FB_ corresponding to the *V*_TG_ where the plot of −(*k*_B_*β*)/*q*, at the subthreshold regime, deviates from a linear response (*cf.*Fig. 4d). The latter deviation is associated with the onset of tunneling injection and, hence, with a bias region where eqn (4) is no longer valid. A detailed explanation of the extraction method can be found elsewhere.^29,30^ It is worth noting that by using the extracted values, eqn (4) describes the experimental data (not shown here) with substantial accuracy within the subthreshold regime. Furthermore, both methods can benefit from dedicated test structures limiting tunneling injection such as the ones suggested elsewhere.^29,30^

**Fig. 5:**
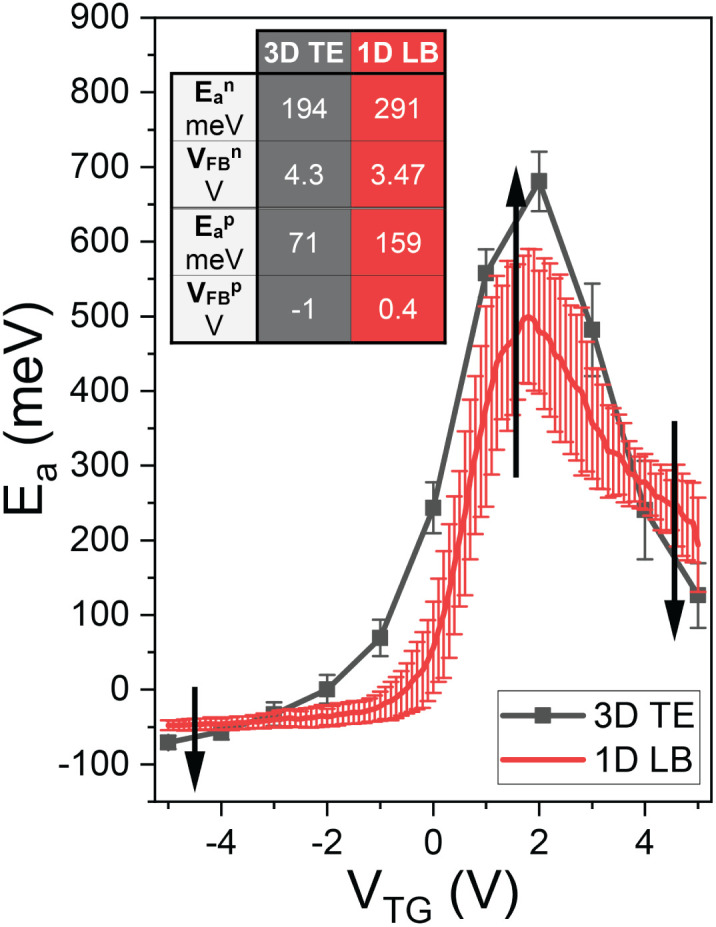
Comparison of the *V*_TG_-dependent activation energies *E*_a_ extracted by 3D TE (grey) and 1D LB (red) theory. The table shows the extracted mean values of four devices and their standard deviation. The black arrows illustrate the difference between 3D TE and 1D LB at specific points of interest.

Finally, comparing the *E*_a_ results obtained by 3D TE and 1D LB allows specific differences between the two models to be analyzed. Fig. 5 shows the *V*_TG_-dependent *E*_a_ data of four devices and their standard deviation. Analyzing these data shows that *E*_a_ extracted with the 3D TE methodology leads to more negative values in strong accumulation as compared to the *E*_a_ extraction with the 1D LB model. In this respect, it needs to be considered that at the interpolation at *V*_D_ = 0 V, the 1D LB model explicitly takes the electrostatic gating into account, with the slope being *β* = −(*qE*_a_)/*k*_B_ + *q*/*k*_B_[*n*_g_(*V*_TG_ − *V*_FB_)], whereas in 3D TE the slope is merely defined by *m* = −*E*_a_/*k*_B_, hence leading to a more accurate *E*_a_ extraction with the 1D LB model. Additionally, the 1D LB model was verified by numerical device simulations on carbon nanotube FETs and NW FETs, proving its applicability for the extraction of the activation energy.^29,30^ Moreover, it needs to be considered that the underlying TE model (*cf.*eqn (1)) is defined assuming the injection from a 3D metal into a 3D semiconductor, whereas in this work, the injection from a metal into a quasi-1D semiconductor (NW) is performed (*cf.*Fig. 1). Therefore, it is expected that the 1D LB theory delivers more accurate *E*_a_ values for quasi-1D NW applications as shown elsewhere where it has been compared to results obtained with a different 3D-based extraction methodology.^29^ Another aspect to consider is the effect of the temperature *T* in eqn (2)*vs.*eqn (5), where in the 3D TE model the temperature is considered quadratically and in the 1D LB model linearly, thus deducing a different TE-related injection tendency of charge carriers compared to temperature.^30^ A convenient check of the obtained results is to sum up the extracted n- and p-branch activation energies to verify if the sum equals the band gap energy of Ge (*E*_g_ = 0.67 eV). Considering the table illustrated in Fig. 5 with mean values of the investigated devices one can obtain a Δ*E* of 405 meV and 220 meV for the 3D TE and 1D LB model, respectively; further highlighting the better suitability of the 1D LB model for NW geometries even beyond the quantum confinement limit. Nevertheless, it needs to be considered that *E*_g_ of quantum confined NWs exhibits higher values compared to that of its bulk counterpart.^4^ Remarkably, at the off-point an *E*_a_ value close to the band gap of Ge is estimated as no electrostatic effects from the gate are considered, further confirming the Fermi level pinning close to the valence band. Finally, Table 1 shows the detailed extracted values of the investigated devices.

**Table 1:** Extracted parameters of the different devices using the 3D TE model and the 1D LB theory. The notation of the table elements is TE/LB

Device	*E* ^n^_a_ (meV)	*V* ^n^_FB_ (V)	*E* ^p^_a_ (meV)	*V* ^p^_FB_ (V)
1	—/283	—/3.9	—/99	—/0.2
2	176/—	5/—	61/—	−1/—
3	198/274	4/3.2	101/90	−1/−0.1
4	208/315	4/3.3	51/287	−1/1.1

## 3 Conclusions

In conclusion, the activation energy *E*_a_ of NW-based Al–Ge–Al SBFETs, using 3D TE and 1D LB theory has been investigated. A comparison of the *V*_TG_-dependent *E*_a_ values of both methods revealed an underestimation of 3D TE in strong accumulation and an overestimation at the intrinsic off-point. Considering the band gap of bulk Ge, a deviation of 405 meV for the 3D TE and 220 meV for the 1D LB model was obtained, hence demonstrating a better suitability of the 1D LB theory for the extraction of an injection activation energy for 1D NW-based SBFETs even beyond quantum confinement. The provided comparison contributes to a better understanding of the extraction of activation energies, which is important for SBFET device optimization.

## Data availability

The data that support the findings of this study are available from the corresponding authors upon reasonable request.

## Author contributions

M. S. and R. B. performed the device fabrication. R. B. conducted the measurements. A. P. conducted the 1D LB investigations. R. B. and A. P. wrote the manuscript. S. B. provided the Ge NWs. M. S. and W. M. W. conceived the project and contributed essentially to the experimental design.

## Conflicts of interest

There are no conflicts to declare.
